# Allergy to soft cannula of insulin pump in diabetic patient

**DOI:** 10.12669/pjms.331.11554

**Published:** 2017

**Authors:** Yu-Min Chen, Hui Huang

**Affiliations:** 1 Yu-Min Chen, MD. Department of Endocrinology and Metabolism, West China Hospital, Sichuan University, Chengdu, Sichuan 610041, China; 2 Hui Huang, PhD. Professor, Department of Endocrinology and Metabolism, West China Hospital, Sichuan University, Chengdu, Sichuan 610041, China

**Keywords:** Allergy, Diabetes, Hyperglycemia, Soft cannula

## Abstract

Insulin pump is a relatively good choice for diabetic patients who require multiple daily injections with wide fluctuations of blood glucose. Patients using insulin pump therapy and still having uncontrolled blood glucose levels for various factors: insulin pump not working properly, insulin instability, insulin autoantibody, insulin allergy, etc. We described a 46-year-old woman with type 2 diabetes and progressive hyperglycemia after switching multiple daily insulin injections to insulin pump, due to allergy to soft cannula of insulin pump.

## INTRODUCTION

Insulin pump (continuous subcutaneous insulin infusion, CSII), is a battery-operated programmable device with basal and bolus insulin infusions over 24h a day to simulate physiological insulin delivery. CSII have the superiority in controlling blood glucose without an increase in hypoglycemic events, compared with multiple daily insulin injections.^1^-^3^ However, still, few patients treated with CSII had poor glucose control for various factors. Here, we report a case of diabetic patient with uncontrolled blood glucose levels due to allergy to soft cannula of insulin pump. To the best of our knowledge so far, no such case has ever been reported.

## CASE REPORT

A 46-year-old woman with seven months duration of diabetes was referred to our hospital because of the wide fluctuation of blood glucose from 2.1mmol/L to 20mmol/L. Prior to admission, lispro, glargine, metformin and acarbose were prescribed successively for controlling patient’s blood glucose. She had no history of allergies.

On hospitalization, her fasting blood glucose was 9.36mmol/L and 2-hour postprandial blood glucose 27.36mmol/L, fasting C-peptide level was 0.106nmol/L(0.48-0.78nmol/L) and 2-hour postprandial C-peptide level 0.218nmol/L(1.34-2.5nmol/L); HbA1c 9.1%; IAA(insulin autoantibody) negative; the function of thyroid, renal and liver were normal. CSII therapy via insulin pump was started with lispro (basal infusion rate of 0.5 units/hour from 7:00 to24:00; 0.3 units/hour from 24:00 to 3:00; 0.4 units/hour from 3:00 to7:00 and mealtime bolus of 4U for each pre-meal). The first day after starting insulin pump, the blood glucose dropped to 8.5mmol/L (the fasting blood glucose) and 8.8~14.2mmol/L(2-hour postprandial blood glucose). Next day, we added the lispro infusion rate and the bolus before meals for better glycemic control. But during the following four days, her blood glucose level continuously rose up to 14.6mmol/L (the fasting blood glucose) and 23.8mmol/L(2-hour postprandial blood glucose) in spite of increasing insulin dosage (Table-I). At the sixth day, as replacing the infusion tube and insulin pump soft cannula, we found the injection site was red, swollen and blistered skin (diameter about 1cm), while the surrounding skin which stuck with adhesive was normal. Then, the injection site was replaced and the previous infusion insulin dosage was kept. The blood glucose was dramatically dropped and the patient had hypoglycemic reaction with post-breakfast glucose 3.6 mmol/L the next day. Nevertheless, the third day after replacing the cannulation site, the patients fasting blood glucose level was 11.8mmol/L and 2-hour postprandial glucose 22.6mmol/L(Table-I). Similarly, the skin at new cannulation site also became red and swollen, but the skin reactions at previous injection site gradually subsided.

**Table-I:** Blood glucose levels and insulin dosage after initiation of insulin pump therapy.

Day	BG (mmol/L) pre- breakfast	BG (mmol/L) post- breakfast	BG (mmol/L) post-lunch	BG (mmol/L) post-dinner	Basal insulin dosage(units/hour)			pre-meal insulin dosage (unit)		

					7:00 -24:00	24:00 -3:00	3:00 -7:00	Breakfast	Lunch	Dinner
1	8.5	11.8	14.2	8.8	0.5	0.3	0.4	4	4	4
2	7.6	9.9	6.8	17.1	0.5	0.3	0.5	4	6	4
3	9.8	11.7	9.4	13.8	0.52	0.3	0.55	7	6	6
4	13.5	13.9	18.0*	17.0	0.56	0.4	0.6	7	6	6
5	14.6	17.1**	9.7	23.8***	0.58	0.5	0.65	7	8	8
6^&^	11.0	13.2	12.3	5.5	0.58	0.5	0.65	7	8	8
7	7.4	3.6	11.6	4.9	0.58	0.5	0.65	8	6	8
8	11.8	22.6	-	-	0.58	0.5	0.65	6	-	-

* Temporarily added 4 U lispro and retested blood glucose (pre-dinner) 13.5 mmol/L;

** Temporarily added 4 U lispro and retested blood glucose (pre-lunch) 10.2 mmol/L;

*** Temporarily added 10 U lispro and retested blood glucose (0:00am) 15.2 mmol/L;

& replaced infusion tube, insulin pump cannula and cannulation site after breakfast.

Hypersensitive reactions in cannulation sites was suspected, and the patient was switched to multiple daily insulin injections regimen (glargine and lispro). The next three days, her blood glucose was dramatically decreased by adjustment of the insulin dosage according to the level of glycemia and no abnormal reaction to injection site had been found. Allergy to soft cannula of insulin pump was immediately considered in this patient. The following days, multiple daily insulin injections regimen was continued and the glycemic control was good (fasting glucose from 5.5 to 6.3mmol/L, 2-hour postprandial glucose from 6.4 to 9.6mmol/L) without hypoglycaemia.

## DISCUSSION

To our knowledge, this case is the first one reported about a diabetic patient on an insulin pump with poor blood glucose control due to allergy to soft cannula of insulin pump.

CSII is one of the options for patients with diabetes requiring multiple daily insulin injections and wide fluctuations of blood glucose.^4^ However, some patients with insulin pump therapy still had poor glucose control which might be caused by many reasons: insulin pump not working properly, insulin instability, insulin secretion deficiency, insulin autoantibody, insulin allergy, etc.^5^ Among them, insulin infusion system malfunction accounts for majority of problems, which often occurred in the syringe, infusion tube and connections, or subcutaneous infusion site, resulting in interruption of insulin flow.^6^ Endermic induration and inflammation in injection site can influence the insulin absorption and change qualities of insulin to some extent.

In general, the above situations can be avoided by double check of the “mini device” or replacing the infusion machinery and injection site. For this patient, IAA negative and poor islet βcell function could not explain the irregular changes in her blood glucose. In addition, she remained hyperglycemic after the infusion tube, cannula and injection site was replaced, which meant the hyperglycemia wasn’t caused by the failure of insulin pump or improper injection position. Anaphylaxis was suspected when the same skin reaction occurred even after switching the soft cannula site. There are previous cases reported that insulin allergy was an important cause that resulted in induration, swelling and redness at injection site.^7^,^8^ But, this patient had had no allergy symptoms since starting insulin therapy seven months ago and still had no swelling or redness at injection site when switched to multiple daily insulin injections in our hospital. All indicated that the patient’s skin reaction of injection site might be related to allergy to the soft cannula of insulin pump and influence the insulin absorption.

In summary, allergy to soft cannula of insulin pump should be considered when patients with CSII therapy have unexplained progressive rise in blood glucose after excluding other possible causes.
